# *Camellia japonica* Flower Extract and the Active Constituent Hyperoside Repair DNA Damage Through FUNDC1-Mediated Mitophagy Pathway for Skin Anti-Aging

**DOI:** 10.3390/antiox14080968

**Published:** 2025-08-06

**Authors:** Hongqi Gao, Jiahui Shi, Guangtao Li, Zhifang Lai, Yan Liu, Chanling Yuan, Wenjie Mei

**Affiliations:** 1 School of Pharmacy, Guangdong Engineering Technology Research Centre of Molecular Probe and Biomedicine Imaging, Guangdong Pharmaceutical University, Guangzhou 510006, China; gaohongqi@lqxgroup.com (H.G.); 2112340121@stu.gdup.edu.cn (J.S.); 2112340143@stu.gdup.edu.cn (Z.L.); 2112240230@stu.gdup.edu.cn (Y.L.); yuanchanling@rubybiotech.cn (C.Y.); 2 Shanghai Forest Cabin Biological-Tech Co., Ltd., Shanghai 201600, China; liguangtao@lqxgroup.com; 3 Guangzhou Ruby Biotechnology Co., Ltd., Guangzhou 510006, China

**Keywords:** *Camellia japonica* flower extract, hyperoside, DNA damage, FUNDC1-mediated mitophagy pathway, skin anti-aging

## Abstract

Skin aging is closely related to mitochondrial dysfunction and cell cycle abnormalities, and developing intervention strategies targeting mitochondrial quality control is an important direction for anti-aging research. In this study, we investigated the anti-aging mechanism of *Camellia japonica* flower (CJF) extract and its active ingredient hyperoside based on a doxorubicin (DOX)-induced endogenous senescence model in human skin fibroblasts (HSFs). LC-MS proteomics analysis revealed that CJF extract and hyperoside specifically activated the FUNDC1-mediated mitochondrial autophagy pathway, significantly ameliorated the DOX-induced decrease in mitochondrial membrane potential and the accumulation of reactive oxygen species (ROS), and alleviated the cellular S-phase blockade and reversed the high expression of senescence-associated β-galactosidase (SA-β-gal). Further studies showed that the two cleared damaged mitochondria by enhancing mitochondrial autophagy and restoring cellular energy metabolism homeostasis while promoting type III collagen and elastin synthesis and repairing the expression of Claudin 1 related to skin barrier function. For the first time, the present study reveals the molecular mechanism of CJF extract in delaying skin aging by regulating the FUNDC1-dependent mitochondrial autophagy pathway, which provides a theoretical basis and a candidate strategy for developing novel anti-aging agents targeting mitochondrial quality control.

## 1. Introduction

As the most significant protective organ in the human body, the aging process of the skin is driven by a combination of endogenous (chronological) and exogenous (environmental) factors [1], leading to structural damage, molecular abnormalities, and accumulation of senescent cells (SCs) [2]. Studies have shown that skin senescent cells can induce dysfunction in neighboring or distal cells through paracrine mechanisms [3]. However, the systemic cascade effects and molecular mechanisms have not been fully elucidated. It has been found that skin senescence may act as a “gas pedal” to promote systemic senescence through multi-organ interactions (e.g., neuroendocrine–immune axis) [4], and the local application of senolytic agents or chemotherapeutic strategies may systematically slow down the aging of the organism [5]. The cells of the nervous, immune, circulatory, and endocrine systems residing in the skin constitute a unique “skin-system” communication network [6]. Recent evidence shows that skin aging is significantly associated with age-related diseases such as metabolic disorders, immune dysregulation and neurodegeneration [3,7]. Developing corresponding anti-skin aging products based on the molecular mechanism of skin aging will promote the rapid development of anti-aging products.

Skin aging mechanisms involve oxidative stress [8], DNA damage, collagen breakdown, epigenetic changes, and microbiome imbalance [9]. Innovative active components and delivery strategies that target and alter key pathways have been studied recently. Ergothioneine (EGT) is a natural antioxidant with powerful anti-inflammatory properties [10,11]. It is widely used in anti-aging skincare [12]. However, EGT product development and application are limited because of its poor stability and low transdermal absorption. Tetrahydrocurcumin (THC) is a hydrogenated metabolite of curcumin [13] which has shown remarkable skin anti-aging potential [14], especially in terms of antioxidant [15] and anti-inflammatory activity [16], promoting collagen synthesis [17] and inhibiting melanin production [18]. However, its application still faces problems such as low transdermal absorption rate, high cost, and unclear mechanism. Autophagy activators, such as Rocaglamide, a rapamycin analog, effectively eliminate SA-beta-Gal-positive cells by inhibiting the activation of autophagosomes through the mTORC1 pathway. However, long-term topical use may inhibit the function of immune cells and cause the side effect of reduced lymphatic infiltration. In addition, niacinamide and retinol can reduce abnormal methylation of age-related genes such as p16INK4a and promote the proliferation of epidermal stem cells [19]. Clinical trials have shown that 5% niacinamide and retinol can improve skin texture and reduce VISIA wrinkle density by 26% [20]. However, using niacinamide and retinol has apparent concentration-dependent side effects, and a concentration of >5% will cause skin redness, itching, stinging, and other discomfort [21]. Some people will have allergic reactions and other side effects, so its broad application is limited [22,23]. Although these widely used skin anti-aging molecules have significant effects, they still have some shortcomings, so their application in skin anti-aging is limited to a certain extent. Therefore, developing novel skin anti-aging molecules with excellent effects, less irritation and side effects, and more apparent mechanisms will broaden this application.

*Camellia japonica* flower (CJF) has a lengthy history of application in China, especially in beauty and anti-aging. Its flowers, leaves, and seeds are widely used in traditional medicine and skincare practices [24]. Li Shizhen, a pharmacist and naturalist in the Ming Dynasty of China, mentioned in the Compendium of Materia Medica that *Camellia japonica* flower has the effect of “clear heat and detoxify, cool blood and stop bleeding” and is often used to treat skin inflammation and repair damage. Modern scientific research has also confirmed that CJF extract is rich in polyphenols, flavonoids, and saponins, which have antioxidant, anti-inflammatory, and collagen synthesis effects, providing a scientific basis for its traditional anti-aging application [25,26,27,28]. In this study, the active components of CJF extract, with excellent anti-aging effects and low toxicity on HSF cells of skin fibroblasts, were screened in vitro. The results showed that the CJF extract contains rich bioactive substances such as hyperoside, eriodictyol, and kaempferol, among which hyperoside can significantly enhance cell viability and repair DNA damage [29,30]. Using a doxorubicin (DOX)-induced endogenous senescence model of HSF cells [31], LC-MS proteomics analysis revealed that CJF extract and hyperoside may effectively improve mitochondrial dysfunction, alleviate S-phase arrest, and reverse age-related phenotype β-galactosidase expression reduction by activating the FUNDC1-mediated mitophagy pathway [32]. In addition, CJF extract and hyperoside significantly promoted type III collagen synthesis and improved skin barrier function. This study revealed the molecular mechanism of CJF extracts against skin aging by regulating the mitophagy pathway, providing a theoretical basis for developing novel anti-aging strategies targeting mitochondrial quality control.

## 2. Materials and Methods

### 2.1. Materials and Chemicals

*Camellia japonica* flowers were harvested in February 2023 from a local Camellia plantation (Dexing, China) [33]. We purchased hyperoside, eriodictyol, kaempferol, ellagic acid, and isoquercitrin reference formulations from Shanghai Macklin Bio-Technology Co., Ltd. (Shanghai, China). Chromatography-grade acetonitrile, methanol, and phosphoric acid were bought from Shanghai Macklin Bio-Technology Co., Ltd. (Shanghai, China). BD Pharmingen™ PI/RNase Staining Buffer was purchased from Becton, Dickinson and Compan (Franklin Lakes, NJ, USA). A mitoROS assay Kit and DAPI Kit were purchased from Beyotime Biotechnology Co., Ltd. (Shanghai, China). MTT and doxorubicin hydrochloride (DOX) were purchased from Fisher Chemical Co. (Waltham, MA, USA). The antibodies of β-actin, GAPDH, HERC2, 53BP1, ATM, FUNDC1, BNIP3L, and LC3B were purchased from Proteintech Co., Ltd. (Wuhan, China), and γH2AX, elastin, Collagen III, and Claudin 1 were purchased from Abcam. (Cambridge, UK).

### 2.2. Analysis of Material Components in Extracts of Camellia japonica Flower

#### 2.2.1. Preparation of CJF Extracts

We weighed 10 g of crushed red *Camellia japonica* flower powder, loaded it into a conical flask, extracted it with 200 mL of 70% ethanol ultrasonic extraction at room temperature for 30 min, and then extracted it with a 60 °C water bath shock for 2 h after cooling and filtration to obtain the camellia extract. The camellia extract was concentrated by a vacuum rotary evaporation evaporator 5 times and then lyophilized by a freeze dryer.

#### 2.2.2. CJF Extract Detection

The 1260 Infinity III LC System (Agilent, Santa Clara, CA, USA) has an autosampler with a thermostat and a WR diode array detector for experimental method development and validation. The data capture and outcomes were examined using the LC Solution 1.0.0.1 analysis software and the equipment. A Wonda Sil C18 column (250 mm × 4.6 mm, 5 μm) (Shimadzu, Kyoto, Japan) was employed with acetonitrile (A) and 0.2% aqueous acetic acid (B) as the mobile phase. The column temperature was maintained at 30 °C, and the total flow rate was set to 1.0 mL/min. The wavelength was established at 360 nm, and the solvent gradient was as follows: 12–18% A for 10 min, 18% A for 10–15 min, 18–32% A for 15–30 min, 32–40% A for 30–40 min, 40–52% A for 40–50 min, and 40% B for a continuous duration of 5 min.

### 2.3. Cell Cultures

HSF cell lines were acquired from the China Center for Type Culture Collection. All cells were cultured in DMEM media (Hyclone, Logan, UT, USA) containing 10% FBS (PAN-Biotech, Aidenbach, Germany) and 1% penicillin/streptomycin (Hyclone, USA) in a humidified environment containing 5% CO_2_ at 37 °C.

### 2.4. Cell Viability Assay

HSF cell lines were seeded in 96-well plates at a density of 5 × 10^3^ cells per well and exposed to different concentrations of CJF extract (0.224–250 μg/L), hyperoside (1.16–74.32 μg/L), eriodictyol (1.16–74.32 μg/L), kaempferol (1.16–74.32 μg/L), ellagic acid (1.16–74.32 μg/L), and isoquercitrin (1.16–74.32 μg/L) for 72 h. An MTT test identified cell viability. The media was substituted with 0.5 mg/mL MTT and incubated for 4 h at 37 °C in a humidified incubator containing 5% CO_2_. The residual MTT solution was removed, and formazan crystals were solubilized using dimethyl sulfoxide (DMSO). Absorbance at 570 nm was quantified with a Multiskan FC microplate reader (Thermo Fisher Scientific, Waltham, MA, USA).

### 2.5. EdU Proliferation Assay

HSF cells were seeded into 6-well culture plates at a density of 5 × 10^4^/well and incubated overnight at 37 °C in a 5% CO_2_ cell culture incubator before being treated with CJF extract (15 μg/L), hyperoside (9.29 μg/L), DOX (0.2 μM), DOX (0.2 μM) with CJF extract (15 μg/L), and DOX (0.2 μM) with hyperoside (9.29 μg/L) for 72 h. Then, the cells were treated with EdU (10 μM) for 2 h. Cells were washed with PBS and fixed with 4% paraformaldehyde for 15 min, and 0.3% Triton X-100 was incubated with permeabilization for 10 min. Next, 500 μL of Click reaction solution was added and incubated in the dark for 30 min, and DAPI stained the cells in the dark for 10 min. Finally, the fluorescent images were taken under a fluorescence microscope (Leica DMI8, Wetzlar, Germany) and analyzed to calculate the EdU positivity rate.

### 2.6. Bio-TEM

The cells were treated with CJF extract (15 μg/L), hyperoside (9.29 μg/L), DOX (0.2 μM), DOX (0.2 μM) with CJF extract (15 μg/L), and DOX (0.2 μM) with hyperoside (9.29 μg/L) for 72 h. After trypsin digestion, the cells were collected and fixed with pre-cooled 2.5% glutaraldehyde and placed at 4 °C for 3 days. Next, the cells were subjected to gradient dehydration in different concentrations of ethanol, after which the cells were sectioned and stained with osmium acid and lead citrate. Finally, the ultrastructure of the cells was visualized on a copper grid of a transmission electron microscope (TECNAI 10, FEI, Hillsboro, OR, USA) [34].

### 2.7. Flow Cytometry Analysis

HSF cells were inoculated at 5 × 10^4^/well in 6-well culture plates overnight and treated with CJF extract (15 μg/L), hyperoside (9.29 μg/L), DOX (0.2 μM), DOX (0.2 μM) with CJF extract (15 μg/L), and DOX (0.2 μM) with hyperoside (9.29 μg/L) for 72 h. The cells were fixed using prechilled 70% ethanol placed at 4 °C for 1 h, and the cells were stained by PI solution (PI/RNase Staining Buffer, BD Pharmingen, Franklin Lakes, NJ, USA) for 15 min to analyze cell cycle distribution. The collected cells were stained by a MitoSOX probe (Mitochondrial Superoxide Assay Kit) purchased from Beyotime Biotechnology Co., Ltd. (Shanghai, China)) for 20 min to test the mitochondrial ROS level. The stained cells were detected by an Epics XL-MCL flow cytometer (Beckman Coulter, Miami, FL, USA).

### 2.8. Immunofluorescence

HSF cells or HaCaT cells were inoculated at 5 × 10^4^/well in 6-well culture plates overnight and treated with CJF extract (15 μg/L), hyperoside (9.29 μg/L), DOX (0.2 μM), DOX (0.2 μM) with CJF extract (15 μg/L), and DOX (0.2 μM) with hyperoside (9.29 μg/L) for 72 h. The cells were fixed with 4% paraformaldehyde for 20 min, permeabilized with 0.5% Triton X-100 for 15 min, and then blocked with 2% BSA for 1 h. The cells were incubated with primary and secondary antibodies at 4 °C for 12 h, respectively, and DAPI stained the nucleus for 5 min. The pictures were captured using the confocal microscope (Zeiss LSM 980, Baden-Württemberg, Germany).

### 2.9. Protein Mass Spectrometry Analysis

HSF cells (5 × 10^5^ cells/plate) were cultured in a 10 cm culture dish. The cells were treated with DOX (0.2 μM), DOX (0.2 μM) with CJF extract (15 μg/L), and DOX (0.2 μM) with hyperoside (9.29 μg/L) for 72 h. Then, the cells were washed with ice-cold PBS 3 times, and lysed in 200 µL of RIPA lysis buffer on ice for 1 h to extract the total protein. The collected proteins were heated in a boiling water bath for 5 min and separated by SDS-PAGE (50 µg protein per lane). The proteins were analyzed by LC-MS by BGI Co., Ltd. (Shenzhen, China) and the intensities and *m*/*z* ratios of ions from MS spectra were used to identify and quantify proteins [35].

### 2.10. Western Blotting

HSF cells were lysed with RIPA lysis solution at 4 °C for 30 min to extract total cellular protein; protein concentration was determined by the BCA method. The protein sample was electrophoresis in 10% SDS-PAGE gel with equal proteins loaded in each lane. After electrophoresis, the separated proteins were transferred to a nitrocellulose membrane and blocked with 5% skimmed milk in TBST buffer for 1 h. The membrane was then incubated overnight at 4 °C with primary antibody at 1:1000 in 5% skimmed milk and then incubated with secondary antibody at 1:2000 for 1 h at room temperature. Protein bands were visualized on the LICOR Odyssey System (Lincoln, NE, USA).

### 2.11. Statistical Analysis

The data obtained were performed using GraphPad Prism 7.04 software (GraphPad, San Diego, CA, USA). The results are presented as the means ± standard error of the mean (SEMs). A two-tailed *t*-test analyzed the difference between two groups, while values were compared among multiple groups using one- and two-way ANOVA, respectively. Dunnett’s multiple comparisons test was applied to analyze the correlation between the control, model and sample groups. Differences were considered statistically significant when *p* < 0.05 (*), *p* < 0.01 (**), *p* < 0.001 (***); ns—not significant.

## 3. Results

### 3.1. Camellia japonica Flower Extract Composition Analysis

**Figure 1:**
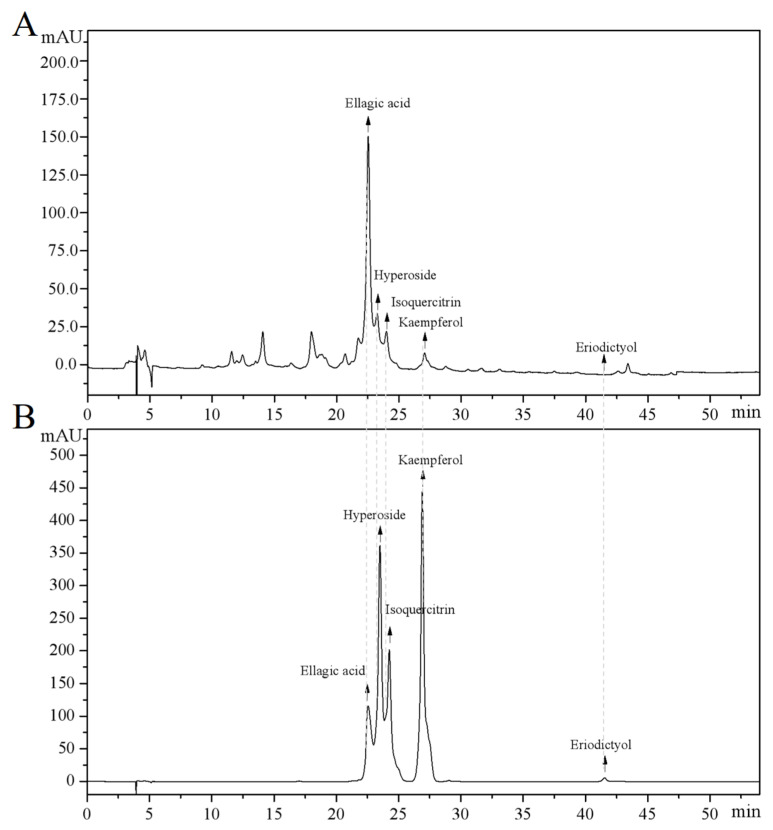
HPLC analysis of major active constituents in CFJ extract. (**A**) HPLC chromatogram of the CFJ extract showing the separation peaks of ellagic acid, hyperoside, isoquercitrin, kaempferol, and eriodictyol. (**B**) HPLC chromatogram of reference standards (1 mg/mL each) with the elution order kaempferol, hyperoside, isoquercitrin, ellagic acid, and eriodictyol.

**Figure 2:**
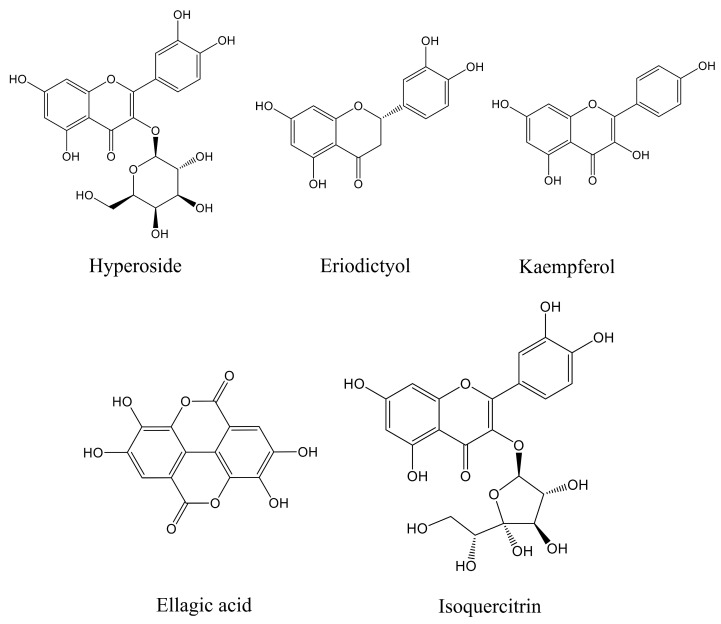
Molecular structures of five active components in CJF extract in this study.

The main components of the prepared CJF extract were analyzed by high-performance liquid chromatography (HPLC), and the results of the HPLC analysis were plotted (see Figure 1). The retention time of each substance in the sample was compared with that of the reference standard to determine the compound species of five substances, which were hyperoside (A), eriodictyol (B), kaempferol (C), ellagic acid (D), and isoquercitrin (E) (Figure S1) [36]. The structural formulae are shown in Figure 2. Method validation was carried out by evaluating the following analytical parameters: linearity, precision, reproducibility, stability, and spiked recovery. The results of precision, reproducibility, stability, and relative standard deviation (RSD) of the spiked recoveries were less than 2%, demonstrating the reproducibility of our results. The results were within the acceptable range, further validating our approach. The above results indicated that the method can be used to analyze CJF extracts. Combined with the results of the linear relationship, the contents of hyperoside, eriodictyol, kaempferol, ellagic acid, and isoquercitrin in the CJF extract were calculated to be 110.99, 5.75, 21.61, 2258.83, and 106.99 ppm, respectively, and the experimental results are shown in Table S1. The results showed that the CJF extract was rich in ellagic acid, hyperoside, and isoquercitrin.

### 3.2. CJF Extract Enhances Cell Viability

**Figure 3:**
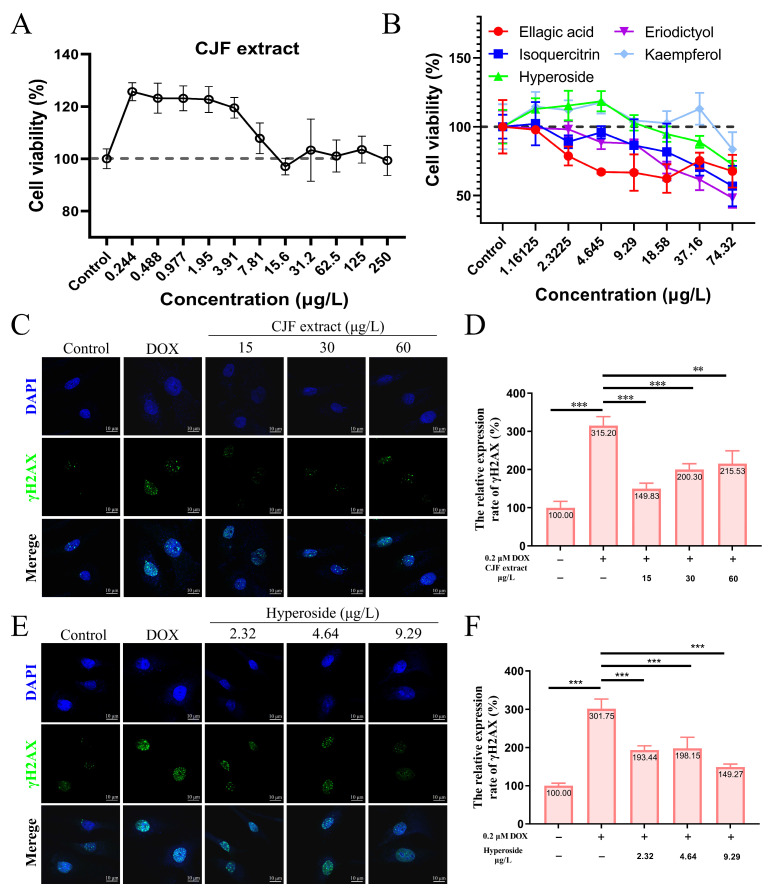
CJF extract and hyperoside promoting DNA damage repair. (**A**) Cell viability of HSF cells after 72 h treatment with CJF extracts. (**B**) Cell viability of HSF cells after 72 h treatment with hyperoside, eriodictyol, kaempferol, ellagic acid, and isoquercitrin. The effects of CJF extract (**C**) and hyperoside (**E**) on the phosphorylation level of γH2AX in the cell nucleus. (**D**) Change in γH2AX foci number in the nucleus for the HSF cells treated with CJF extract (15, 30, and 60 μg/L) for 72 h. (**F**) Change in γH2AX foci number in the nucleus for the HSF cells treated with hyperoside (2.32, 4.64, and 9.29 μg/L) for 72 h. The data were represented graphically as means ± SEM. Statistical significance was determined by one-way ANOVA. Values significantly differed from the control group at significance levels of ** *p* < 0.01, and *** *p* < 0.001.

To screen the effective concentration range of CJF extract for promoting cell proliferation, the effect of different concentrations of CJF on HSF cell viability was examined using the methylthiazolyltetrazolium (MTT) method [37]. The results showed that treatment with CJF at concentrations of 0.224–7.81 μg/L significantly enhanced HSF cell viability (>100%) compared to the blank control group, indicating a pro-proliferative effect within this concentration range. And after treatment with CJF at 15.6–250 μg/L, the HSF cell viability remained around 100%, suggesting no significant impact on the cells within this higher concentration range (Figure 3A). Further evaluation of the effects of the main monomer components of CJF (hyperoside, eriodictyol, kaempferol, ellagic acid, and isoquercitrin) revealed that ellagic acid at >2.32 μg/L, eriodictyol and isoquercitrin decreased cell viability at >4.65 μg/L, indicating that these active components exhibited some degree of influence to HSF cells. In contrast, hyperoside and kaempferol significantly promoted cell proliferation at <9.29 μg/L. Given the low content of kaempferol in CJF (HPLC quantification <0.05% *w*/*w*), the high abundance fraction of hyperoside was selected for this study to investigate its sub-sequent mechanism.

### 3.3. CJF Extract Promotes DNA Damage Repair

DOX is a widely used chemotherapy drug that induces DNA double-strand breaks (DSBs) by embedding DNA double strands and inhibiting topoisomerase II activity. Constant DNA damage triggers cell cycle stagnation, ultimately leading to cell senescence [38]. In addition, DOX metabolizes in the cell to generate many reactive oxygen species (ROS), causing mitochondrial dysfunction and oxidative stress, further aggravating the damage of DNA, proteins, and lipids, and promoting the expression of age-related phenotypes [39]. Therefore, DOX is often used to construct endogenous senescence models. As shown in Figure 3C, after 0.2 μM DOX treatment, the number of γH2AX foci in HSF cells increased significantly, and the expression level increased to 315.20% (*p* < 0.01), indicating that DOX successfully induced the DNA damage model. In contrast, treatments with 15, 30, and 60 μg/L CJF extract significantly reduced the amount of DOX-induced γH2AX foci. The expression levels decreased to 149.83%, 200.30%, and 215.53%, respectively (Figure 3D). Of these, 15 μg/L CJF extract showed the best DNA damage repair effect. Further studies showed that after hyperoside treatment of DOX-induced HSF cells at concentrations of 2.32, 4.64, and 9.29 μg/L, the number of γH2AX foci decreased significantly (Figure 3E). The expression levels decreased to 193.44%, 198.15%, and 149.27%, respectively (Figure 3F). Among them, 9.29 μg/L hyperoside had the most significant repair effect. These results indicate that CJF extract is rich in components with DNA damage repair activity, and hyperoside may be one of its key active components. This study provides the experimental basis for applying camellia extract and its active components in anti-aging.

### 3.4. CJF Extract Regulating Cell Phase Arrest Improves Cell Proliferation

**Figure 4:**
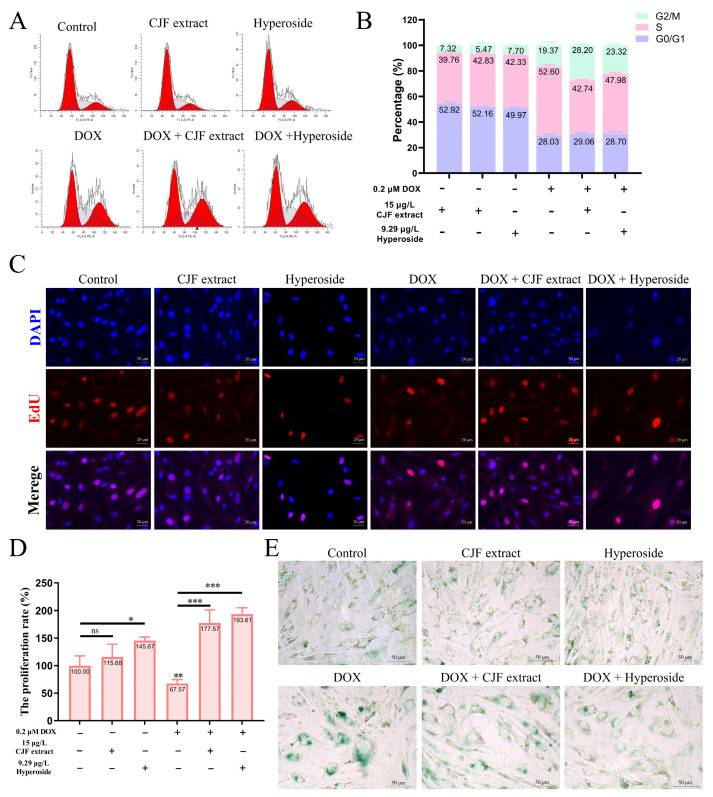
Regulation of cell phase arrest with CJF extract and hyperoside improves cell proliferation. (**A**) The cell cycle distribution of the HSF cells after treatment with CJF extract and hyperoside for 72 h. (**B**) The percentage of the cell cycle of the HSF cells changed upon the addition of CJF extract and hyperoside. (**C**) The EdU assay evaluated the proliferation of HSF cells after treatment with CJF extract and hyperoside for 72 h. (**D**) The proliferation rate was determined by the ratio of cells with EdU-positive staining. (**E**) Detection of cellular senescence by SA-β-Gal staining. The data were represented graphically as means ± SEM. Statistical significance was determined by one-way ANOVA. Values significantly differed from the control group at significance levels of * *p* < 0.05, ** *p* < 0.01, and *** *p* < 0.001, ns—not significant.

One of the main characteristics of cell senescence is irreversible arrest of the cell cycle [40]. This study further evaluated the regulatory effects of CJF extract and its active component, hyperoside, on the cell cycle (Figure 4A). The normal HSF cell treatment with 15 μg/L CJF extract and 9.29 μg/L hyperoside did not significantly change cell cycle distribution. However, HSF cells showed significant S-phase arrest (52.60%) and G2/M phase arrest (19.73%) after 0.2 μM DOX treatment for 72 h, indicating that DOX successfully induced cell cycle arrest in the aging model (Figure 4B). Of note, treatment with 15 μg/L CJF extract significantly alleviated DOX-induced S-phase arrest (proportion decreased to 42.74%), but the G2/M phase arrest increased to 28.20%. Similarly, 9.29 μg/L hyperoside treatment significantly reduced the proportion of the S-phase arrest (47.96%), while the G2/M phase arrest increased to 23.32%. The S phase is a critical stage of DNA synthesis, and its arrest is usually triggered by replication fork stagnation or DNA damage, resulting in double-strand breaks or single-strand nicks. If the damage is repaired within the permitted time, the cells can restart through checkpoints and enter the G2/M phase [41]. These results suggest that CJF extract and hyperoside may promote some S-phase arrest cells to enter the G2/M phase by repairing DNA damage, thereby partially restoring the cell cycle process. In summary, CJF extract and hyperoside alleviate DOX-induced S-phase arrest and promote cell transition to the G2/M phase by regulating cell cycle checkpoints, suggesting their potential anti-aging effects.

Furthermore, the loss of cell proliferation capacity is a critical hallmark of cellular senescence [42]. This study used EdU staining assays to investigate the regulatory effects of CJF extract and hyperoside on the proliferation rate of HSF cells. The results demonstrated that compared to DAPI-labeled cell counts, the 15 μg/L CJF extract and 9.29 μg/L hyperoside treatment groups exhibited a significant increase in EdU-positive cells, with proliferation rates rising to 115.68% (*p* > 0.05) and 145.67% (*p* < 0.05), respectively. These findings suggest that CJF extract and hyperoside markedly enhance HSF cell proliferation. As shown in Figure 4C, it is found that the DOX-treated group displayed a significant reduction in EdU-positive cells compared to the blank control group, with the proliferation rate declining to 67.57% (*p* < 0.01), confirming the successful construction of a senescence model characterized by diminished proliferative capacity. Notably, in the DOX-induced senescence model, both 15 μg/L CJF extract and 9.29 μg/L hyperoside treatment groups showed a significant increase in EdU-positive cells relative to the model control group, with proliferation rates elevated to 177.57% (*p* < 0.001) and 193.61% (*p* < 0.001), respectively (Figure 4D).

In addition, the results of β-galactosidase (SA-β-gal) staining showed that the SA-β-gal staining signal was expressed at a low level in the HSF cells of the blank control group, the CJF extract-treated group, and the hyperoside-treated group, suggesting that normal physiological state or single-component treatments did not activate the cellular senescence-associated phenotype significantly (Figure 4E). However, after induction by DOX, the intensity of SA-β-gal staining in HSF cells was significantly increased (both the proportion of positive cells and the depth of staining increased), indicating that DOX successfully established a cellular senescence model. Notably, when combined with CJF extract or hyperoside treatment based on DOX induction, the SA-β-gal staining signal was significantly weakened, and both the proportion of positive cells and the staining intensity were significantly lower than those in the treatment group with DOX alone. This result suggests that CJF extract and hyperoside can effectively inhibit the DOX-induced upregulation of β-galactosidase activity and thus antagonize the senescence process of HSF cells. Based on these experimental findings, we propose that CJF extract and hyperoside can regulate multiple mechanisms such as cell cycle block, promote DNA damage repair, restore proliferative capacity, and inhibit the expression of aging markers, showing significant anti-skin aging potential.

### 3.5. CJF Extract Induces Autophagy

**Figure 5:**
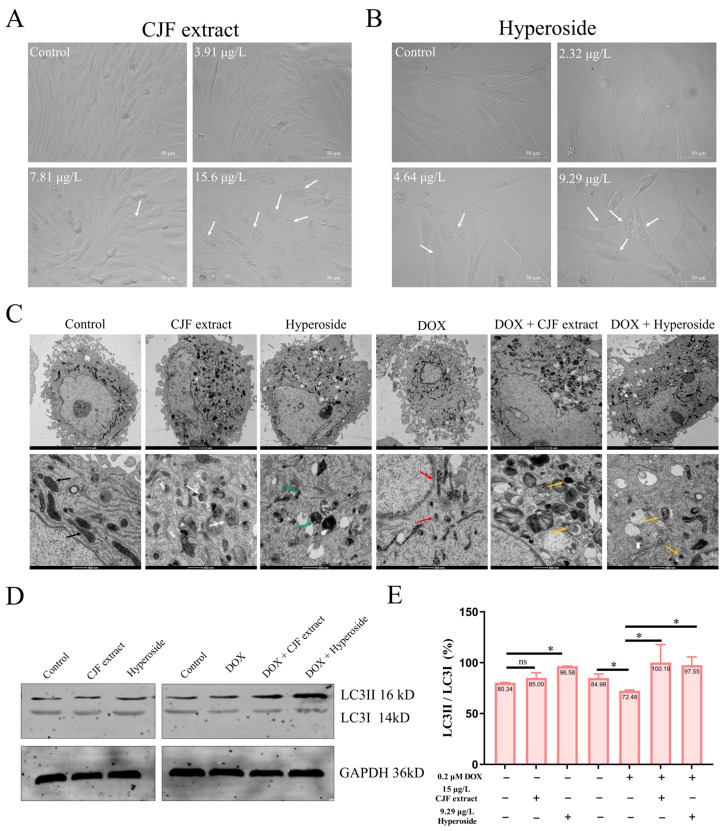
Autophagy induced by CJF extract and hyperoside. (**A**) The morphological changes in HSF cells without and with CJF extract for 72 h. (**B**) The morphological changes in HSF cells without and with hyperoside for 72 h. (**C**) Bio-TEM imaging of HSF cell autophagy induction by CJF extract and hyperoside. The black arrows refer to well-defined cristae, the white arrows refer to multilamellar bodies, the green arrows refer to mitochondrial debris, the red arrows refer to cristae loss, the yellow arrows refer to mitochondrial fragments. Scale bar: 2 μm (top); Scale bar: 500 μm (below). (**D**) Changes in the autophagy marker protein expression of LC3II and LC3I at the protein level. HSF cells were treated with CJF extract and hyperoside for 72 h. (**E**) LC3II/LC3I protein band ratio histogram. The data were represented graphically as means ± SEM. Statistical significance was determined by one-way ANOVA. Values significantly differed from the control group at significance levels of * *p* < 0.05, ns—not significant.

Our previous findings demonstrated that CJF extract and hyperoside exhibit potent DNA damage repair activity, effectively alleviating DOX-induced cell cycle arrest in senescent HSF cells and restoring their proliferative capacity, indicative of anti-senescence potential. We systematically analyzed cellular morphology and ultrastructural changes using inverted microscopy (Leica DMI1, Wetzlar, Germany) and transmission electron microscopy (TEM) (FEI Tecnai™, Hillsboro, OR, USA) following CJF extract or hyperoside treatment to elucidate the mechanistic basis further. As depicted in Figure 5A, inverted microscopy imaging revealed that HSF cells treated with 15 μg/L CJF extract (but not at lower concentrations of 3.75 or 7.5 μg/L) displayed prominent cytoplasmic vacuolization (white arrows), a morphological hallmark of autophagic activation. It was further demonstrated that 9.29 μg/L hyperoside (the maximally effective concentration) induced the formation of double-membrane autophagosomes (white arrowheads) in HSF cells (Figure 5B). In contrast, no ultrastructural features were observed at suboptimal concentrations (2.32 or 4.64 μg/L). These observations suggest that CJF extract and hyperoside mitigate cellular damage through autophagy induction, potentially underlying their anti-senescence efficacy [43].

As shown in Figure 5C, untreated HSF cells exhibited intact organelles, including mitochondria with well-defined cristae (black arrows), spherical lysosomes (Lys), and elongated endoplasmic reticulum (ER) structures. Strikingly, treatment with 15 μg/L CJF extract or 9.29 μg/L hyperoside induced two distinct autophagic phenotypes: (i) CJF extract-treated cells displayed a 2.3-fold increase in mitochondria with electron-dense matrices (*p* < 0.01 vs. control), accompanied by the formation of multilamellar bodies (MLBs, white arrows), a hallmark of autophagic activation; and (ii) hyperoside-treated cells showed abundant double-membrane autophagosomes (APs) containing mitochondrial debris (green arrows), indicative of selective mitophagy. In contrast, DOX-induced senescent HSF cells (0.2 μM) exhibited severe mitochondrial degeneration, characterized by cristae loss (red arrows) and a 68% reduction in intact mitochondria (*p* < 0.001). Remarkably, CJF extract (15 μg/L) partially restored mitochondrial quantity (1.4-fold increase, *p* < 0.05) but triggered extensive MLB accumulation, whereas hyperoside (9.29 μg/L) increased not only mitochondrial number (1.6-fold, *p* < 0.01) but also promoted autophagic clearance, as evidenced by AP-encapsulated mitochondrial fragments (inset, yellow arrows). These data strongly suggest that hyperoside enhances mitochondrial quality control via mitophagy, while CJF extract may primarily activate bulk autophagy to alleviate senescence-associated damage.

LC3B (microtubule-associated protein 1 light chain 3 beta), a core autophagy marker, is critical in monitoring autophagic activity through its post-translational modification dynamics. During autophagy initiation, the LC3B precursor (pro-LC3B) is proteolytically cleaved to generate LC3-I, which subsequently conjugates with phosphatidylethanolamine (PE) to form the membrane-bound LC3-II isoform. The lipidated LC3-II is integrated explicitly into autophagosomal membranes, serving as a hallmark of autophagosome formation and maturation. Consequently, the LC3-II/LC3-I ratio is widely utilized as a quantitative indicator of autophagic flux, with elevated ratios reflecting enhanced autophagosome biogenesis and increased autophagic activity [44]. To evaluate the autophagy-inducing effects of CJF extract and hyperoside, we performed Western blot analysis to quantify LC3 isoforms in both standard and DOX-induced senescent HSF cells. In normal HSF cells, CJF extract and hyperoside treatment induced a mild upregulation of LC3-II expression without significant alteration in LC3-I levels (Figure 5D). Notably, hyperoside treatment elicited a marked increase in the LC3-II/LC3-I ratio, suggesting its potent capacity to stimulate autophagosome formation. In DOX-induced senescent HSF cells, LC3-I and LC3-II expression levels were substantially elevated compared to the control group. However, despite increased autophagosome accumulation, the LC3-II/LC3-I ratio in senescent cells was reduced to 72.46%, indicative of impaired autophagic flux. Strikingly, CJF extract and hyperoside treatment significantly restored the LC3-II/LC3-I ratio to 100.19% and 97.55%, respectively, in senescent HSF cells (Figure 5E). These data demonstrate that both interventions effectively rescued autophagic dysfunction in cellular senescence, potentially through reactivating autophagosome–lysosome fusion or restoring autophagic flux, thereby highlighting their therapeutic potential in counteracting age-related autophagy decline.

### 3.6. CJF Extract Repaired Mitochondrial Dysfunction

**Figure 6:**
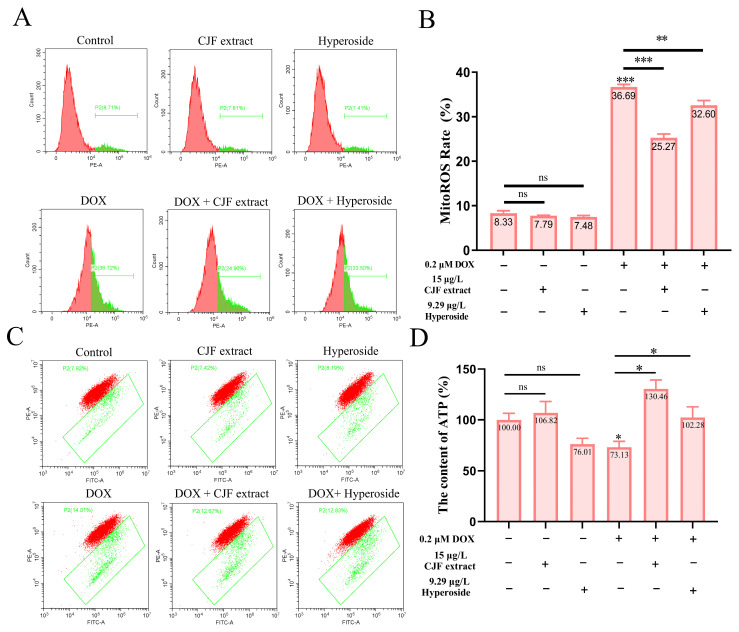
Repair of mitochondrial function through mitophagy induced by CJF extract and hyperoside. (**A**) The MitoROS generation changes in HSF cells without and with CJF extract for 72 h. (**B**) MitoROS ratio histogram. (**C**) Changes in mitochondrial membrane potential by CJF extract and hyperoside. (**D**) Changes in ATP content of HSF cells without and with CJF extract and hyperoside for 72 h. The data are represented graphically as means ± SEM. Statistical significance was determined by one-way ANOVA. Values significantly differed from the control group at significance levels of * *p* < 0.05, ** *p* < 0.01, and *** *p* < 0.001, ns means not significant.

Based on the previous findings that CJF extract and hyperoside treatment significantly increased mitochondrial quantity in DOX-induced senescent HSF cells, we further investigated their effects on mitochondrial dysfunction. To assess mitochondrial reactive oxygen species (mitoROS) levels, we employed the mitoSOX probe [44]. As shown in Figure 6A, neither CJF extract nor hyperoside treatment altered mitoROS levels in normal HSF cells compared to the untreated control. In contrast, DOX-induced senescent HSF cells exhibited a significant increase in mitoROS levels (36.69%), indicating elevated oxidative stress in senescent cells. Treatment with CJF extract and hyperoside notably reduced mitoROS levels to 25.27% and 32.60%, respectively (Figure 6B), suggesting their potential to mitigate mitochondrial oxidative damage.

To evaluate mitochondrial membrane potential (ΔΨm), we utilized the JC-1 probe [39]. Figure 6C demonstrates that neither CJF extract nor hyperoside treatment affected ΔΨm in normal HSF cells. However, DOX-induced senescent HSF cells displayed a marked increase in ΔΨm dissipation (36.69%), indicative of mitochondrial membrane damage and functional impairment. Treatment with CJF extract and hyperoside significantly attenuated ΔΨm dissipation, reducing the proportion of cells with depolarized mitochondria to 25.27% and 32.60%, respectively, highlighting their protective effects on mitochondrial membrane integrity. Given the central role of mitochondria in ATP production [34], we further measured cellular ATP levels. As depicted in Figure 6D, CJF extract treatment did not significantly alter ATP production in normal HSF cells, while hyperoside caused a slight, non-significant reduction. In DOX-induced senescent HSF cells, ATP production was significantly decreased to 73.13% of control levels, reflecting impaired mitochondrial energy metabolism. Strikingly, treatment with CJF extract and hyperoside restored ATP production to 130.46% and 102.28%, respectively, demonstrating their ability to enhance mitochondrial bioenergetic function in senescent cells.

**Figure 7:**
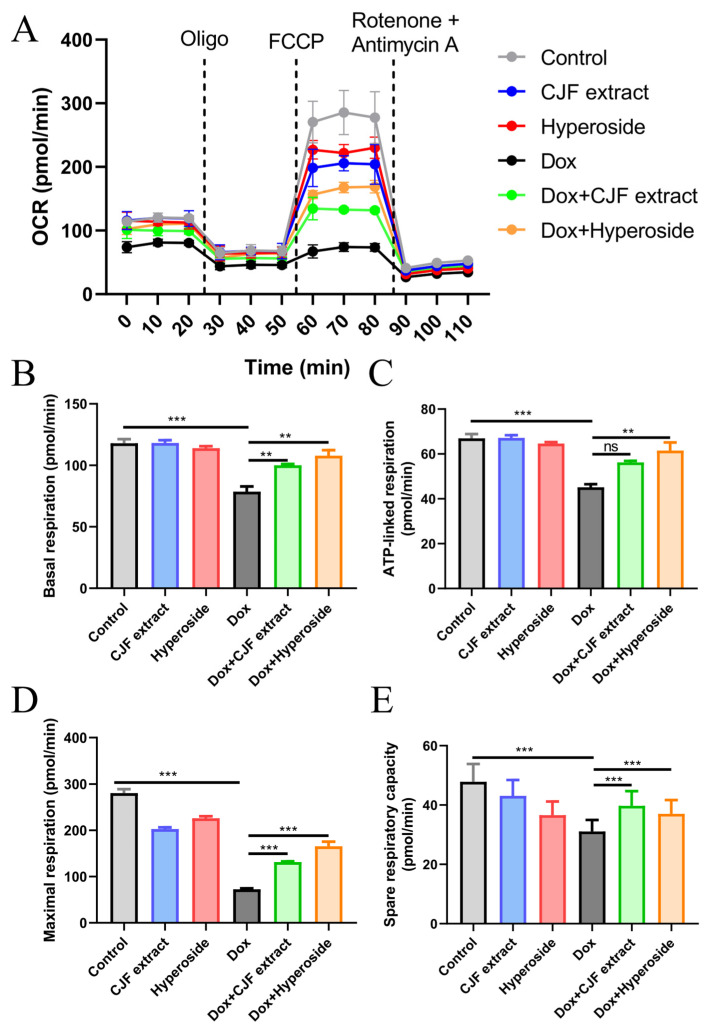
Mitochondrial function assessed by Seahorse Energy Metabolism Analysis. (**A**) Mitochondrial oxygen consumption rate (OCR) was monitored using a Seahorse metabolic analyzer. HSF cells in different treatment groups (control, CJF extract, hyperoside, DOX, DOX with CJF extract, and DOX with hyperoside), in response to the addition of 1 μM oligomycin (Oligo) at 20 min, 5 μM FCCP at 50 min, and 1 μM rotenone + 1 μM antimycin at 80 min, were recorded. (**B**) Basal respiration, (**C**) ATP-linked respiration, (**D**) maximal respiration, and (**E**) spare respiratory capacity in HSF cells were quantified. The data are represented graphically as means ± SEM. Statistical significance was determined by one-way ANOVA. Values significantly differed from the control group at significance levels of ** *p* < 0.01 and *** *p* < 0.001, ns means not significant.

As assessed by the Seahorse Energy Metabolism Analysis System, 0.2 μM DOX treatment for 72 h was found to significantly inhibit key respiratory parameters of HSF cells (Figure 7A), including decreasing basal respiration (Figure 7B), ATP-linked respiration (Figure 7C), maximal respiration (Figure 7D), and residual respiratory capacity (Figure 7E), as compared with the control group. The results confirmed that DOX led to mitochondrial failure through a triple synergistic mechanism, specifically downregulating FUNDC1-mediated mitophagy and hindering the clearance of damaged mitochondria; the quinone structure may inhibit ETC complex I/III activity. In addition, its ROS-induced burst destroys mitochondrial integrity, which ultimately triggers OXPHOS dysfunction and an energy metabolism crisis. In addition, compared with the Dox-treated group, CJF extract enhanced basal, ATP-linked, maximal, and residual respiratory capacities by 27.3%, 24.5%, 81.3%, and 27.7%, respectively. Hyperoside exhibited a more substantial restorative effect, with the corresponding parameters elevated by 37.2%, 36.1%, 128.2%, and 19.1%, respectively, thereby restoring maximal respiration to near-physiological levels. Mechanistic studies revealed that the common protective pathway of the two relied on FUNDC1 expression to mediate locked mitophagy, thereby clearing damaged mitochondria and reversing impairments in ATP synthesis, which is consistent with the previous protein expression changes. Notably, the hyperformal recovery of maximal respiration induced by hyperoside, with an increase of 128.2%, suggests a unique multi-target potentiation mechanism that may inhibit glycolytic excursion through the downregulation of HIF-1α, in addition to the shared autophagy-activating pathway. These findings indicate that CJF extract and hyperoside ameliorate multiple aspects of mitochondrial dysfunction, including oxidative stress, membrane potential dissipation, and bioenergetic deficits, potentially contributing to their anti-senescence effects.

### 3.7. CJF Extract Activated FUNDC1-Mediated Mitophagy Pathway

**Figure 8:**
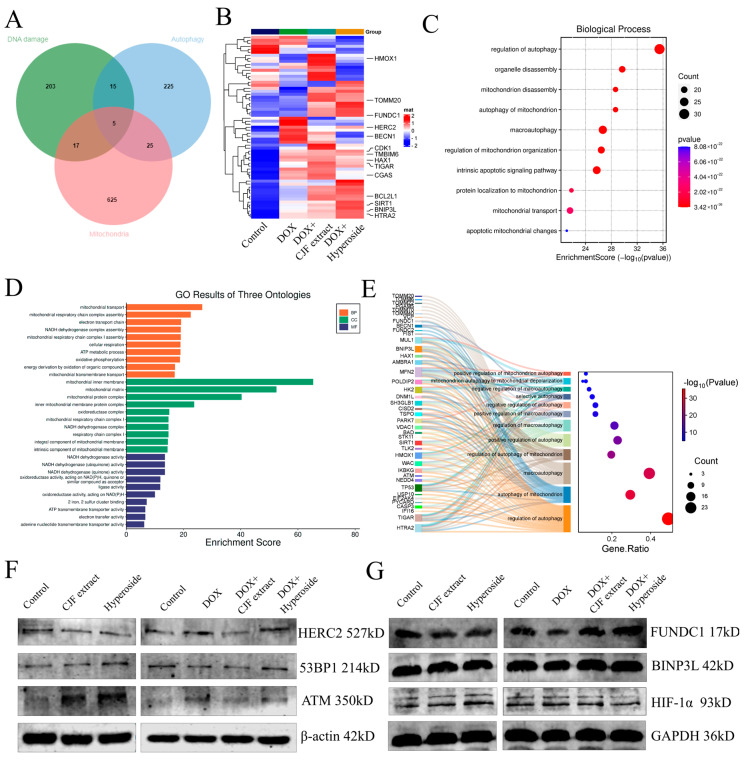
Proteomics analysis and signaling pathway validation of CJF extract and hyperoside. (**A**) Comparisons of DNA damage-related proteins, autophagy-related proteins, and mitochondrial function-related proteins. (**B**) Heat map showing the abundance of DNA damage-related proteins, autophagy-related proteins, and mitochondrial function-related proteins in HSF cells in different treatment groups (control, DOX, DOX with CJF extract, and DOX with hyperoside). (**C**) GO functional enrichment analysis map demonstrates biological processes associated with mitochondrial autophagy. (**D**) Functional categories of enriched proteins. (**E**) Sankey diagram analysis reveals core regulatory proteins of the mitochondrial autophagy pathway. (**F**) The DNA damage repair-related protein expression level changes in 53PB1, ATM, and HERC2 induced by CJF extract and hyperoside. (**G**) The mitophagy-related protein expression level changes in FUNDC1, BINP3L, and HIF-1α induced by CJF extract and hyperoside.

In this study, we systematically analyzed the mechanism of CJF extract and hyperoside in DNA damage repair, mitochondrial function regulation, and autophagy by proteomics technology. The proteomic analysis of the four groups of experimental samples (blank control group, model control group, CJF extract-treated group, and hyperoside-treated group) identified a total of >2000 proteins/group, and the quality of the data was verified by standardized quality control (Figure S2). Based on differential expression protein screening, 240 DNA damage-related proteins, 270 autophagy-related proteins, and 672 mitochondrial function-related proteins were obtained, and 62 coregulated proteins were identified after cross-tabulation analysis (Figure 8A). Among them, HTRA2, HERC2, SIRT1, FUNDC1, and BNIP3L showed the most significant changes in protein expression. HERC2 was significantly upregulated in the DOX-induced injury model (** *p* < 0.01), whereas CJF extract could partially reverse its expression and hyperoside treatment (Figure 8B). According to GO functional enrichment analysis, the above 62 core proteins were mainly enriched in autophagy regulation (including mitophagy and macroautophagy) and mitochondrial function-related biological processes (Figure 8C). Further multidimensional annotation (biological process BP, cellular component CC, and molecular function MF) of differentially expressed genes (DEGs) revealed that the CJF extract and hyperoside-treated groups were significantly enriched in terms of mitochondrial energy metabolism, NADH/ATP synthesis, and mitochondrial autophagy pathways (Figure 8D). Specifically, the two maintained mitochondrial network integrity by activating mitophagy to selectively remove dysfunctional mitochondria and regulate mitochondrial dynamic homeostasis (fusion/disintegration), protein localization, and transport. Sankey diagram analysis further revealed that FUNDC1 and BNIP3L are the core regulatory nodes of the mitophagy pathway (Figure 8E). This study systematically elucidates the molecular pathways of CJF extract and its active component hyperoside, which may play a protective role in cells through a double synergistic mechanism. Firstly, they selectively remove dysfunctional mitochondria by activating the FUNDC1-dependent mitochondrial autophagy pathway, thereby reducing the accumulation of reactive oxygen species (ROS) and alleviating mitochondrial membrane potential dissipation. Secondly, they can significantly improve the localization and transport efficiency of the mitochondrial membrane proteins FUNDC1 and BNIP3L and ensure the function of the electron transport chain and energy metabolism homeostasis.

HERC2 (HECT and RLD Domain-Containing E3 Ubiquitin Protein Ligase 2) is an E3 ubiquitin ligase that regulates DNA damage repair, cell cycle progression, and maintenance of genome stability, mainly by modulating ubiquitination modifications of target proteins. Further studies have shown that HERC2 expression was upregulated in DOX-induced senescent cells, whereas its expression was downregulated to be comparable to that of the blank group in cells treated with CJF extract and hyperoside. However, the expression of ATM and 53BP1, key proteins related to DNA damage repair, was not significantly different after DOX, CJF extract, and hyperoside treatment (Figure 8F). Notably, subsequent treatment of DOX-injured cells with either CJF extract or hyperoside was able to specifically reverse the downregulation of FUNDC1 and significantly upregulate its protein expression, while having no significant effect on the protein levels of BNIP3L, but with weak downregulation of HIF-1α (Figure 8G). The results indicated that CJF extract and hypericum glycoside mainly repaired DNA damage by promoting mitophagy.

### 3.8. CJF Extract Repaired Impaired Skin Barrier

**Figure 9:**
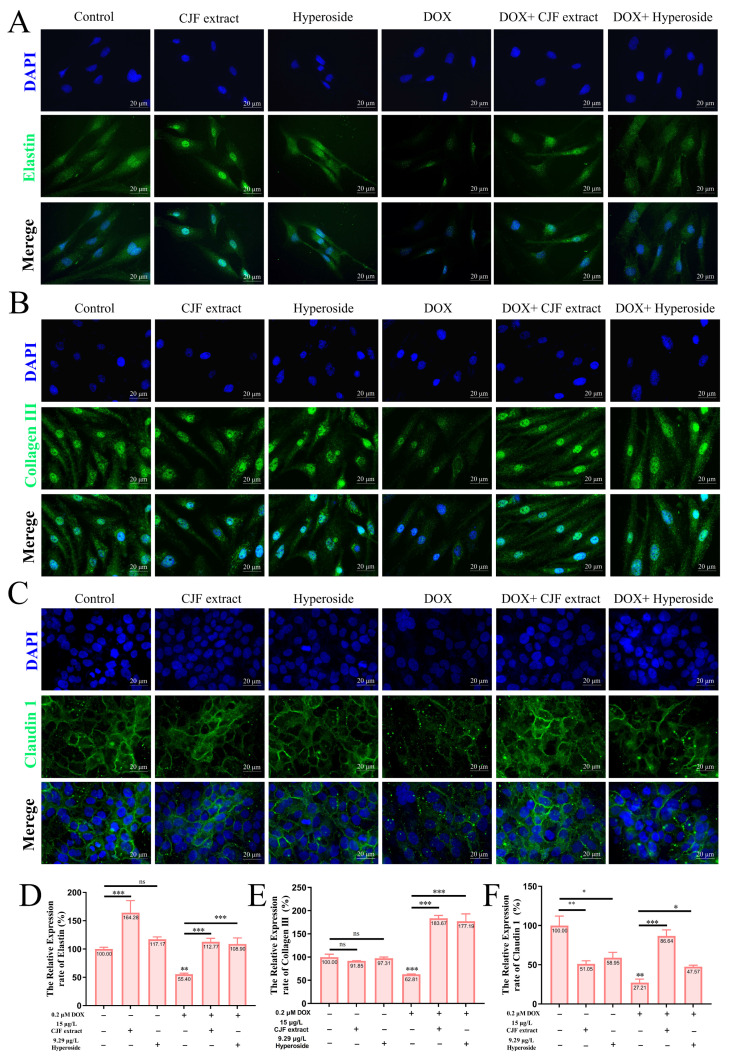
Repair of damaged skin barrier by mitochondrial autophagy with CJF extract and hyperoside. Expression levels of elastin (**A**), Collagen III (**B**), and Claudin 1 (**C**) after 72 h of treatment with CJF extract and hyperoside. Quantitative analysis of the relative expression rates of elastin (**D**), Collagen III (**E**), and Claudin 1 (**F**). The data were represented graphically as means ± SEM. Statistical significance was determined by one-way ANOVA. Values significantly differed from the control group at significance levels of * *p* < 0.05, ** *p* < 0.01, and *** *p* < 0.001, ns means not significant.

This study investigated the repairing effects of CJF extract and hyperoside on DOX-induced skin barrier damage. As shown in Figure 9A,B, the expression of elastin and Collagen III in the CJF extract-treated group alone and the hyperoside-treated group alone compared with the blank group was not significantly different. However, the expression of elastin and Collagen III was significantly reduced in DOX-induced HSF senescent cells compared to the blank control group (*** *p* < 0.001), suggesting that the loss of extracellular matrix (ECM) components in the senescent cells resulted in impaired skin barrier function [45]. Nevertheless, the expression of elastin and collagen type III in HSF cells was significantly restored (*** *p* < 0.001) after treatment with CJF extract and hyperoside, suggesting that the two were effective in promoting the remodeling of the ECM in the dermis and enhancing the structural integrity and elasticity of the skin (Figure 9D,E). As shown in Figure 9C, it is observed that there were no significant differences in the expression of claudin-1 in HaCaT cells in the CJF extract-treated group alone and the hyperoside-treated group alone compared with the blank group. Nonetheless, the tight junction protein Claudin-1 expression in DOX-induced HaCaT keratinocytes was also significantly downregulated (** *p* < 0.01), further validating the successful construction of the DOX-induced skin barrier damage model. Notably, CJF extract and hyperoside treatment significantly upregulated the expression of Claudin-1 in HaCaT cells (* *p* < 0.05), suggesting that it could effectively repair the barrier function of the epidermal layer (Figure 9F) [46]. In summary, CJF extract and hyperoside markedly enhance skin barrier function via a dual mechanism that stimulates the expression of elastin and type III collagen in HSF cells, thereby fortifying the structural integrity of the dermis, and they upregulate Claudin-1 expression in HaCaT cells to restore epidermal barrier function. These *results* suggest that CJF extract and hyperoside significantly ameliorate DOX-induced skin barrier damage by synergistically regulating dermal ECM remodeling and epidermal intercellular junctions, providing a potential molecular strategy for skin repair and treatment of related diseases.

## 4. Discussion

Skin aging, a phenotypic hallmark of organismal senescence, is driven by mitochondrial dysfunction, accumulation of DNA damage, and cell cycle arrest [4]. While autophagy-mediated mitochondrial quality control represents an emerging frontier in anti-aging research [2], synthetic drugs face limitations in terms of cytotoxicity and target specificity. Natural plant extracts offer multi-target advantages for delaying skin aging [3]. However, the anti-senescence mechanisms of CJF extract in fibroblasts, particularly mitophagy regulation, remain uncharacterized. This complex process involves multifactorial synergy, including sleep deprivation, which accelerates aging through the inhibition of collagen synthesis, activation of MMPs (reducing collagen density), epidermal lipid loss (increasing transepidermal water loss), and dysregulation of the HPA/HPG axis (elevated cortisol, lowered testosterone), creating an inflammatory–metabolic vicious cycle. Concurrently, metabolic/endocrine imbalances (including ENO1-mediated glycolytic reprogramming, which causes ATP deficiency and chronic cortisol elevation) impair fibroblast function, which is compounded by nutritional deficits (zinc/vitamin D3) that weaken antioxidant defences and microcirculatory decline, exacerbating dermal ischemia. Molecularly, NF-κB-mediated chronic inflammation suppresses collagen synthesis while promoting MMP secretion; UV-induced ROS cause DNA damage (CPDs) and mitochondrial dysfunction; and senescent cell-derived SASP factors (IL-6/MMPs/TGF-β) propagate senescence via paracrine signaling. Integrated interventions should thus target inflammation (e.g., IL-17 blockade), metabolism (e.g., ENO1 inhibition), nutrition, and sleep (7–9 h/day) to establish multilayered protection.

**Figure 10:**
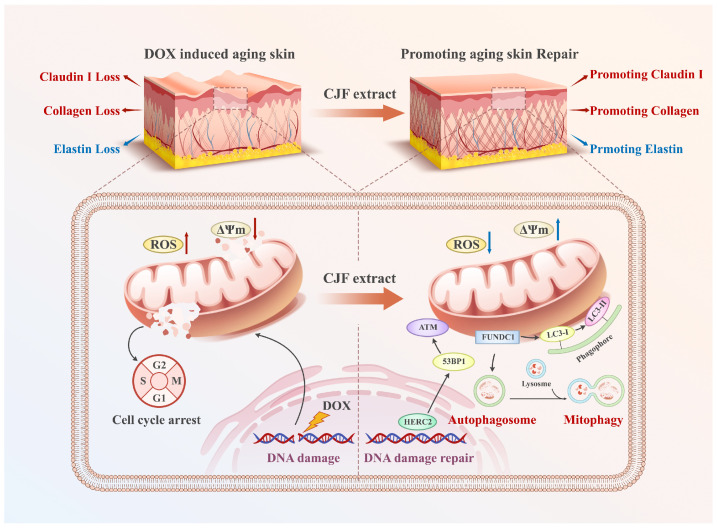
CJF extract and its active constituent hyperoside attenuate DNA damage via the FUNDC1-mediated mitophagy pathway and restore the dysregulated expression of type III collagen, elastin, and Claudin 1 in the DOX-induced skin aging model.

By establishing the DOX-induced senescence model in human skin fibroblast (HSF) cells, this study elucidates for the first time the molecular mechanism by which CJF extract and its principal active compound hyperoside exert anti-aging effects through FUNDC1-mediated mitophagy. MTT assays demonstrated that hyperoside significantly reversed senescence-associated proliferative suppression, which correlated strongly with mitochondrial membrane potential restoration and a 52% reduction in γH2AX foci. These findings collectively suggest that hyperoside synergistically restores mitochondrial functionality and genomic stability to counteract aging phenotypes. Flow cytometry revealed a 29% decrease in G1-phase-arrested cells (*p* < 0.05), confirming its regulatory role in cell cycle re-activation. At the functional level, the 1.7-fold upregulation of type III collagen and 41% increase in Claudin-1 expression mechanistically substantiate the capacity of CJF extract to enhance skin structural integrity and barrier function. Diverging from prior studies emphasizing singular antioxidant pathways, this work identifies FUNDC1 as a pivotal regulatory node in mitophagy. This discovery explains the multidimensional anti-aging effects of CJF extract (e.g., concurrent mitochondrial repair and collagen synthesis). It provides novel insights into natural product-mediated senescence delay through a “mitochondrial–epigenetic” crosstalk network. Compared to the mechanism of green tea polyphenols reported by Chong et al. [47], CJF extract demonstrated superior DNA damage repair capacity (approximately 20% greater reduction in γH2AX foci), potentially attributable to structural modifications unique to hyperoside. This study systematically deciphers the multilayered mechanisms through which CJF extract antagonizes skin aging via the FUNDC1–mitophagy axis. Further studies found that the above mechanisms synergically reversed the age-related degradation of type III collagen and abnormal expression of barrier function protein, providing theoretical support for the clinical transformation potential of CJF extract and hyperoside in anti-aging and oxidative skin damage (Figure 10). These findings provide an innovative theoretical framework for precision anti-aging strategies driven by natural products while establishing an experimental foundation for developing efficacious and safe skin rejuvenation interventions.

In addition to the proven mechanisms of mitophagy and DNA repair, the anti-aging effect of chrysin on skin may be achieved through a multidimensional synergistic pathway. Its downregulation of HIF-1α and reduction in β-galactosidase expression is consistent with the inhibition of senescence-associated secretory phenotype (SASP) effect, which, given the signaling crossover between HIF-1α and NF-κB, may be achieved through inhibition of the NF-κB pathway to reduce the release of IL-6 and other pro-inflammatory factors by inhibiting the NF-κB pathway, improving the micro-inflammatory environment of the skin. The significantly elevated expression of type III collagen and elastin suggests the potential for epigenetic remodeling, which needs to be combined with the PathwayAge model to analyze fibroblast differentiation genes (e.g., COL3A1 promoter methylation) to validate the reversal effect of the “accelerated epigenetic age”. In addition, the reestablishment of barrier proteins, such as Claudin-1, reflects the functional rejuvenation of keratinocytes, which may be associated with the activation of stem cell paracrine secretion (e.g., MSC-EV delivery system) and mitophagy induced by the increase in FUNDC1 protein expression. The elevation of ATP synthesis and downregulation of HIF-1α suggest that it inhibits glycolytic reprogramming (the Warburg effect) and blocks the ENO1-SASP axis through an “energy–antioxidant” dual-acting mechanism, resulting in deep metabolism–inflammation homeostasis regulation.

In the current study, the core pivotal role of FUNDC1 has not been verified as necessary by gene editing (KO/KI), and functional resolution of its phosphorylation modification sites (Ser13/Tyr18) is lacking. Furthermore, the specific pathways (e.g., PHD2/VHL-dependent) of HIF-1α downregulation by hyperoside, as well as the activation mechanism of FUNDC1, have not been elucidated. Moreover, the causal association between ATP restoration and the activation of DNA repair enzymes (PARP1/DNA-PKcs) and collagen synthase lacked direct evidence. In addition, the mechanistic differences between the DOX model and natural aging have not been discussed, and the changes in FUNDC1-mediated mitophagy pathway activity in human aging skin also remain to be verified.

## 5. Conclusions

This research utilized HSF cells to evaluate anti-aging constituents from CJF extracts. Hyperoside is essential for improving cell viability and reversing the aging phenotype. In a DOX-induced skin cell aging model, CJF extracts and hyperoside were observed to repair and mitigate DNA damage, restore mitochondrial membrane potential, decrease mitochondrial ROS levels, relieve S-phase arrest, enhance cell proliferation, reverse the expression of the aging marker β-galactosidase, stimulate the synthesis of elastin and type III collagen, and restore skin barrier functionality. These actions improve mitochondrial dysfunction by modulating the FUNDC1-mediated mitophagy signaling pathway. This work is the first investigation to elucidate a molecular mechanism connecting the anti-aging effects of CJF extracts to mitochondrial quality control, offering novel insights into the formulation of anti-aging medicines aimed at mitochondria.

## Data Availability

Data will be made available on request.

## Supplementary Materials

The following supporting information can be downloaded at: https://www.mdpi.com/article/10.3390/antiox14080968/s1, Figure S1: The HPLC analysis graphs of hyperoside (A), isoquercitrin (B), ellagic acid (C), kaempferol (D), and eriodictyol (E); Table S1: The contents of hyperoside, eriodictyol, kaempferol, ellagic acid, and isoquercitrin in CJF extracts; Figure S2: Proteomics analysis of different experimental groups.

## Abbreviations

The following abbreviations are used in this manuscript:

ATP	Adenosine triphosphate
BNIP3L	BCL2 interacting protein 3-like
BP	Biological process
CC	Cellular component
CJF	*Camellia japonica* flower
DEGs	Differentially expressed genes
DOX	Doxorubicin hydrochloride
DSBs	DNA double-strand breaks
ECM	Extracellular matrix
FUNDC1	FUN14 domain-containing protein 1
GO	Gene ontology
HERC2	HECT and RLD domain-containing E3 ubiquitin protein ligase 2
HPLC	High-performance liquid chromatography
HTRA2	HtrA serine peptidase 2
MF	Molecular function
MTT	Methylthiazole tetrazole
NADH	Nicotinamide adenine dinucleotide
ROS	Reactive oxygen species
SIRT1	Sirtuin 1
TEM	Transmission electron microscopy
