# Correction: Gao et al. *Camellia japonica* Flower Extract and the Active Constituent Hyperoside Repair DNA Damage Through FUNDC1-Mediated Mitophagy Pathway for Skin Anti-Aging. *Antioxidants* 2025, *14*, 968

**DOI:** 10.3390/antiox15050620

**Published:** 2026-05-14

**Authors:** Hongqi Gao, Jiahui Shi, Guangtao Li, Zhifang Lai, Yan Liu, Chanling Yuan, Wenjie Mei

**Affiliations:** 1 School of Pharmacy, Guangdong Engineering Technology Research Centre of Molecular Probe and Biomedicine Imaging, Guangdong Pharmaceutical University, Guangzhou 510006, China; gaohongqi@lqxgroup.com (H.G.); 2112340121@stu.gdup.edu.cn (J.S.); 2112340143@stu.gdup.edu.cn (Z.L.); 2112240230@stu.gdup.edu.cn (Y.L.); yuanchanling@rubybiotech.cn (C.Y.); 2 Shanghai Forest Cabin Biological-Tech Co., Ltd., Shanghai 201600, China; liguangtao@lqxgroup.com; 3 Guangzhou Ruby Biotechnology Co., Ltd., Guangzhou 510006, China

In the original publication [1], there was an error: “concentration unit: μg/mL” should be replaced with “concentration unit: μg/L”, and “hypericin” revised as “hyperoside” is intended solely to correct a typographical error. This modification does not affect the scientific content, data interpretation, results, or conclusions of the manuscript, and no other issues are involved.

A correction has been made to 3.2. CJF Extract Enhances Cell Viability:

To screen the effective concentration range of CJF extract for promoting cell proliferation, the effect of different concentrations of CJF on HSF cell viability was examined using the methylthiazolyltetrazolium (MTT) method [37]. The results showed that treatment with CJF at concentrations of 0.224–7.81 μg/L significantly enhanced HSF cell viability (>100%) compared to the blank control group, indicating a pro-proliferative effect within this concentration range. And after treatment with CJF at 15.6–250 μg/L, the HSF cell viability remained around 100%, suggesting no significant impact on the cells within this higher concentration range (Figure 3A). Further evaluation of the effects of the main monomer components of CJF (hyperoside, eriodictyol, kaempferol, ellagic acid, and isoquercitrin) revealed that ellagic acid at >2.32 µg/L, eriodictyol and isoquercitrin decreased cell viability at >4.65 µg/L, indicating that these active components exhibited some degree of influence to HSF cells. In contrast, hyperoside and kaempferol significantly promoted cell proliferation at <9.29 µg/L. Given the low content of kaempferol in CJF (HPLC quantification <0.05% *w*/*w*), the high abundance fraction of hyperoside was selected for this study to investigate its sub-sequent mechanism.

The authors state that the scientific conclusions are unaffected. These modifications ensure more rigorous expression, more precise alignment with the data, and avoidance of overstatement. This correction was approved by the Academic Editor. The original publication has also been updated.

## References

[1] Gao H., Shi J., Li G., Lai Z., Liu Y., Yuan C., Mei W. (2025). *Camellia japonica* Flower Extract and the Active Constituent Hyperoside Repair DNA Damage Through FUNDC1-Mediated Mitophagy Pathway for Skin Anti-Aging. Antioxidants.

