# Supplementary material for: Camellia japonica Flower Extract and the Active Constituent Hyperoside Repair DNA Damage Through FUNDC1-Mediated Mitophagy Pathway for Skin Anti-Aging
Source: Antioxidants (Basel). 2025 Aug 6;14(8):968. doi: 10.3390/antiox14080968 (PMC12383142; doi:10.3390/antiox14080968)
Supplement: Supplementary file 1 [file antioxidants-14-00968-s001.zip › antioxidants-3675943-supplementary.pdf]

Supporting Information

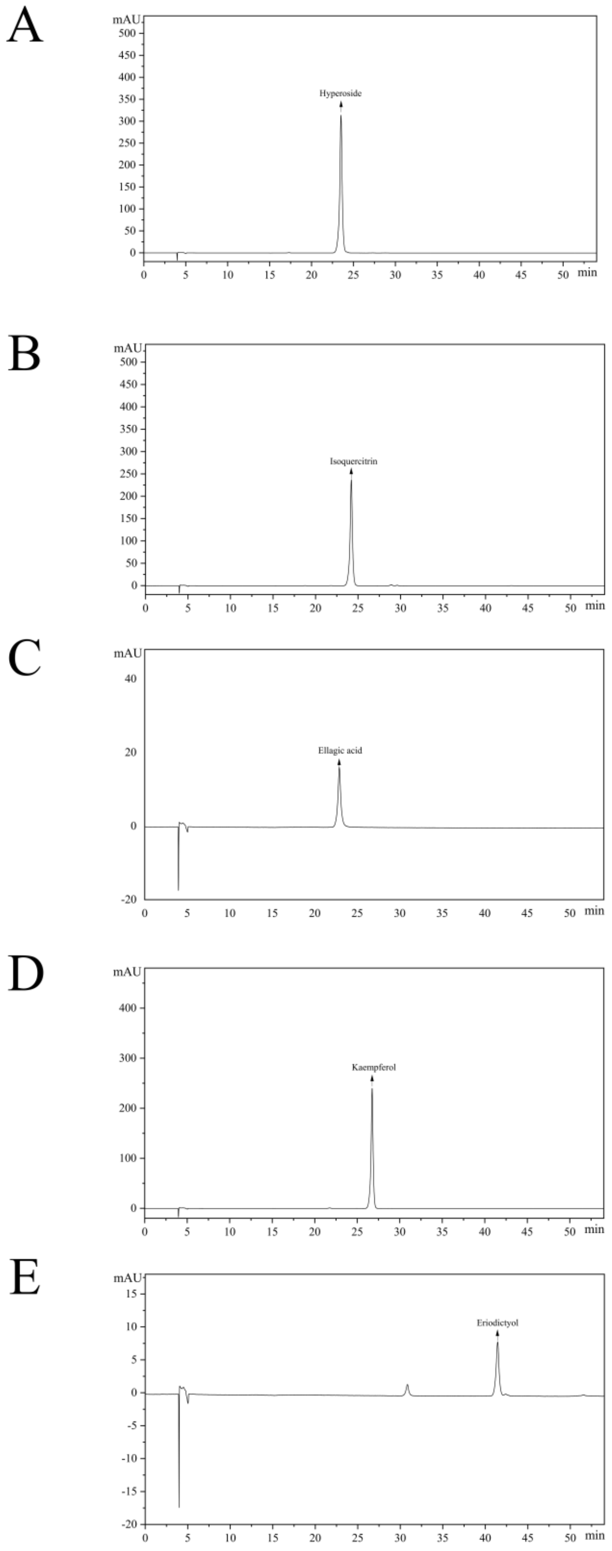

Figure S1. The HPLC analysis graphs of hyperoside (A), isoquercitrin (B), ellagic acid (C), kaempferol (D), and eriodictyol (E).

Table S1. The contents of hyperoside, eriodictyol, kaempferol, ellagic acid and isoquercitrin in CJF extracts.

Sample	Content (ppm)
Hyperoside	110.99
Eriodictyol	5.75
Kaempferol	21.61
Ellagic acid	2258.83
Isoquercitrin	106.99

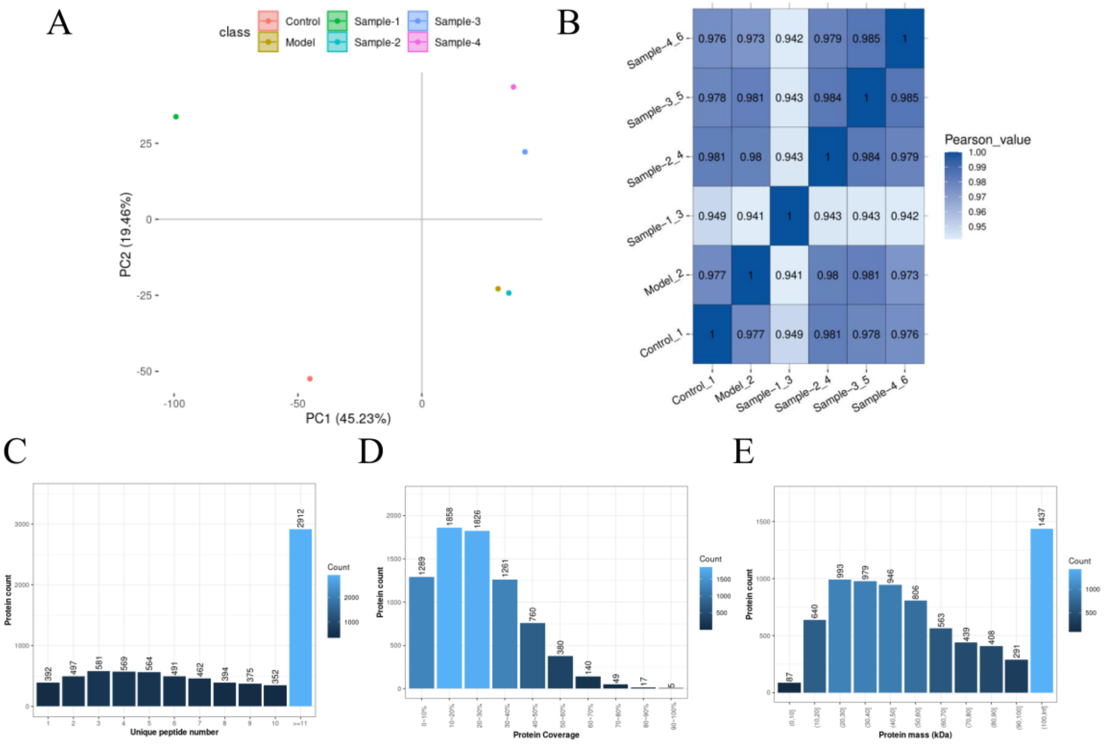

Figure S2. Proteomics analysis of different experimental groups. (A) Principal component analysis (PCA) plot illustrating global proteomic differences among different sample groups. (B) Pearson correlation heatmap showing the correlation of protein expression among different sample groups. The color scale represents Pearson correlation coefficients, with darker blue indicating higher correlations. The high

correlation among replicates suggests strong reproducibility of the proteomic data, while differences between groups indicate potential variations in protein expression. (C) Distribution of identified proteins based on the number of unique peptides. (D) Protein coverage distribution across all identified proteins. (E) Distribution of identified proteins based on molecular weight (kDa).
